# Supplementary material for: Psychosocial career preoccupation and organisational commitment at higher educational institutions in Ghana: The role of workplace friendship
Source: PLoS One. 2026 Mar 2;21(3):e0340460. doi: 10.1371/journal.pone.0340460 (PMC12952573; doi:10.1371/journal.pone.0340460)
Supplement: S1 Data — (DOCX) [file pone.0340460.s001.docx]

Supporting information (Appendix 1, Data)
