# Supplementary material for: Psychosocial career preoccupation and organisational commitment at higher educational institutions in Ghana: The role of workplace friendship
Source: PLoS One. 2026 Mar 2;21(3):e0340460. doi: 10.1371/journal.pone.0340460 (PMC12952573; doi:10.1371/journal.pone.0340460)
Supplement: Attachment — Submitted filename: Response to reviewers.docx [file pone.0340460.s004.docx]

Response letter

Dear editor,

| **S/N** | **Reviewer(s)** | **Responses** |
| --- | --- | --- |
| 1 | The manuscript identifies multiple gaps in the literature, such as the multidimensionality of psychosocial career preoccupations, the varied dimensions of organisational commitment, and broader contextual challenges in Ghanaian higher education. However, the study empirically addresses only a subset of these (specifically the mediating role of workplace friendship). While this focused approach is valid, the authors should acknowledge more explicitly that many of the gaps remain unaddressed and position their study as an initial step, with future research needed to cover the wider scope | This has been addressed in the study. Refer to page 17 under the limitation of the study. |
| 2 | Though Author(s) clearly stated that Average Variance Extracted (AVE) values for Organisational Commitment (0.363), Psychosocial Career Preoccupation (0.489), and Workplace Friendship (0.477) are below the 0.5 threshold. However, they did not state it as a limitation. | This has been addressed in page 17 as well. |
| 3 | They discussion also, lack more empirical evidence and how it aligned with the studies' findings. | This has also been addressed. |
| 4 | The use of only one institution limits generalizability across public institutions in Ghana. Including more institution may give diverse results and better understanding on the interactions among Organizational Commitment, Psychosocial Career Preoccupation and Workplace Friendship across institutions. | This has been captured as a limitation in the study which further studies could consider. |
| 5 | You may want to consider control variables such as age and gender to ensure a truer relationship between the main variables of interest ie. Psychosocial career Preoccupation, Occupational Commitment and Workplace Friendship. | This could not be addressed because the main concern of the study was the mediating role of workplace friendship. This has been well discussed in the write-up. The control variables as suggested could be considered in further studies. Again, the control variables age and gender have been extensively considered by authors; Ferreira et al. 2024. |
| 6 | Provide citations that support the statement made in page 10, line 8. | This has been addressed in page 10, line 8 |
| 7 | Provide citations on the research gap mentioned in page 10, line 18. | This has been addressed in page 11, line 18 |
